# A modified hand washing method for resource limited settings

**DOI:** 10.3389/fpubh.2022.965853

**Published:** 2022-08-04

**Authors:** Samreen Sarwar, Javed Muhammad, Faheem Shahzad

**Affiliations:** ^1^Health Security Partners, Lahore, Pakistan; ^2^Department of Microbiology, University of Haripur, Haripur, Pakistan; ^3^Department of Immunology, University of Health Sciences, Lahore, Pakistan

**Keywords:** handwashing, laboratory, resource-limited settings, implementation, compliance

## Abstract

The Good Microbiological Practices & Procedures (GMPP) is the most significant risk control measure as per the fourth edition of the WHO laboratory biosafety manual. Among GMPP, one of the best practices is hand washing. WHO and other public health agencies have published several guidance documents on hand washing, that describe closing the tap using a disposable paper towel/tissue paper at the end of hand washing as one of the critical steps. In resource-limited settings, where disposable paper towels cannot be provided at all times, the staff is left with ambiguous instructions on how to close the tap. In this paper, a modified hand washing method is documented that doesn't necessitate the use of disposable paper towels. In this method, both hands and faucets remain in contact with soap for at least 40–60 s. The method was validated by the use of Glo Germ. A survey questionnaire was also designed and conducted for the lab staff (*n* = 12) of the two laboratories, where this method was implemented, to assess whether this hand washing method brought any improvement in their hand washing practices and implementation. All (100%) of the survey respondents reported that this method of hand washing is more applicable and implementable than the WHO-recommended hand washing technique. Eighty three percentage reported that this modified method of hand washing raised their hand washing compliance. The authors suggest that this hand washing method can be used in resource-limited laboratory settings as an effective GMPP to ensure infection control.

## Introduction

Biosafety practices in laboratories are based on the principle of containing biological agents to reduce the risk of laboratory-acquired infections (LAIs) by preventing exposure of laboratory personnel and the outside environment (1). The fourth edition of the WHO laboratory biosafety manual prescribes core requirements that must be implemented in all laboratories regardless of the level of work that is being done in that lab. These core requirements can effectively control risks encountered in the majority of clinical and diagnostic laboratory activities (2). Activities that may pose a higher risk and cannot be mitigated by the core requirements, should be assessed using a risk assessment framework. Once risk is assessed, relevant, and sustainable risk control measures that are commensurate with the risks should be implemented (2). The Good Microbiological Practices & Procedures (GMPP) is the most significant risk control measure to be incorporated into the core requirements. GMPP refers to a set of standard operating procedures and practices, often written in a code of practice, that applies to all forms of biological agent activity. Among GMPP, one of the best practices to be followed by all laboratory staff is hand hygiene (2). Hand hygiene is a simple and inexpensive practice to avoid LAIs. There is sufficient evidence to prove that hand hygiene alone, when properly implemented, can significantly decrease the risk of LAIs in laboratories (3). Public health agencies are, therefore, emphasizing the importance of improving hand hygiene keeping in view the increasing severity of infections, and the emergence of multi-drug resistant (MDR) pathogens (3). Handwashing is a cost-effective component of hand hygiene. A handwashing sink, which is an engineering control, is also one of the core requirements for a laboratory (2).

The World Health Organization (WHO) and the U.S. Centers for Disease Control and Prevention (US CDC), have produced several resources, including posters (4) and videos^1^ that explain why, when, and how hands should be washed in a laboratory or hospital setting. These training materials break down the process of hand washing into 9 to 11 steps that can be completed with a hand/elbow-operated tap. In this method, closing the tap with a disposable paper towel/tissue paper at the end of handwashing is one of the critical steps. Hand washing sinks are either manual (hand/elbow-operated taps) or automated. Automated hand washing is an expensive option that is not feasible in most facilities because of its electricity requirements and sophisticated design. Manual hand washing is the only option left, which necessitates the use of tissue paper or paper towels, which adds to the cost of handwashing (5).

The majority of laboratories in resource-constrained countries like Pakistan have hand-operated taps. A few new laboratories are using elbow-operated taps, but closing elbow-operated taps while wearing street clothes or exposing bare elbow to the tap after removing PPE and before leaving the lab exposes street clothes/skin to contamination from the dirty faucet, which laboratory workers can bring home or outside the lab. Sinks with foot pedals are also not a viable option in all Pakistani laboratories because they are 10 times more expensive, take up more space in the lab, require specific design fits, and need changing and reinstalling water piping systems (6). The design is only suitable for new constructions and cannot be applied to existing systems without modifying the tap and/or the connections, which are frequently located inside the walls. Some designs necessitate altering the basin or its pillar (6). The majority of the labs, particularly those in the public sector, cannot afford to provide paper towels to their employees regularly. When paper towels aren't available, staff are left with ambiguous instructions on how to close the tap. This is a critical gap that we have addressed in Pakistan in two national antimicrobial resistance (AMR) reference laboratories. This paper aims to explain how this was addressed so that the global community can benefit from it.

The Fleming Fund is helping low- and middle-income countries in tackling AMR through its country grants (7). Health Security Partners (HSP) is part of a consortium that is supporting the Fleming Fund in Pakistan. As one of the project's first steps, two BSL-2 veterinary sector laboratories were identified as AMR reference laboratories. One of the objectives that the Fleming fund tried to accomplish in this grant was to develop and strengthen their biorisk management system using the new WHO risk assessment-based approach (2) following the biosafety program management monograph (8). In this monograph, step 3 in the biosafety program management cycle (8) is implementation through the development and communication of standard operating procedures for the safe work practices in these laboratories. Handwashing is the most critical and effective practice in biosafety and biosecurity. Therefore, to accommodate the unavailability of disposable paper towels, biosafety experts implemented a modified way of handwashing for the lab professionals working in these laboratories. We believe that this method can be employed in all research and diagnostic laboratories that deal with biological agents in a variety of fields.

## Materials and methods

**Figure 1:**
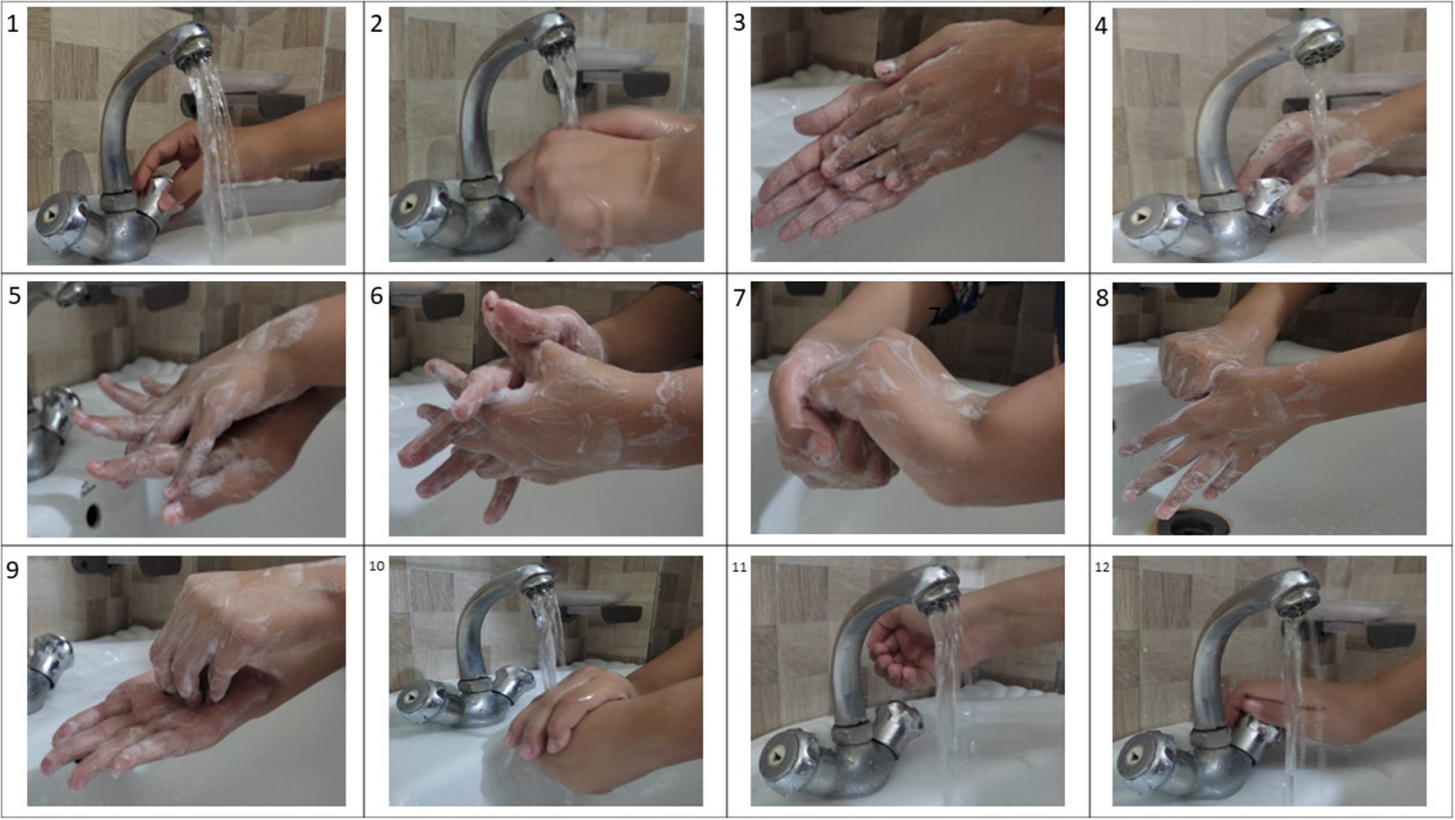
The steps for the modified hand washing method: 1. open tap, 2. wet hands with water, 3. apply enough soap on hand and rub hands palm to palm to make enough foam, 4. close the tap using the foamed hand evenly applying the foam all over the tap handle/faucet, 5. rub the area of the hand by placing right palm over left dorsum with interlaced fingers and vice versa, 6. rub hands palm to palm with fingers interlaced, 7. rub back of fingers with opposing palms with fingers interlocked, 8. perform rotational rubbing of left thumb clasped in right palm and vice versa, 9. perform rotational rubbing, backward and forward with clasped fingers of the right hand in left palm and vice versa, 10. open the tap and rinse hands with water, 11. rinse the faucet with enough water to remove any foam on the faucet, 12. now close the tap with clean hands.

Due to the use of regular hand-operated taps and the unavailability of disposable paper towels in all sections of these laboratories, a modified method of handwashing was implemented by biosafety experts who were helping the two veterinary laboratories, designated as the AMR reference laboratories, in the implementation of a biosafety program. This experimental study only included the laboratory workers from the two veterinary sector AMR reference labs that implemented this modified handwashing method. In this method the following steps were initiated (Figure 1).

1. Open the tap
2. Wet hands with water
3. Apply enough soap on hand and rub hands palm to palm to make enough foam
4. Close the tap using the foamed hand evenly applying the foam all over the tap handle/faucet
5. Rub the area of the hand by placing right palm over left dorsum with interlaced fingers and vice versa
6. Rub hands palm to palm with fingers interlaced
7. Rub back of fingers with opposing palms with fingers interlocked
8. Perform rotational rubbing of left thumb clasped in right palm and vice versa
9. Perform rotational rubbing, backward and forward with clasped fingers of the right hand in the left palm and vice versa
10. Open the tap and rinse hands with water
11. Rinse the faucet with enough water to remove any foam on the faucet
12. Now close the tap with clean hands

In this method, both hands and faucets should be in contact with soap for at least 40–60 s.

The method was validated by the use of Glo Germ (Glo Germ^TM^, USA). Glo Germ is a visual tool for teaching proper handwashing and aseptic techniques. In this method, a nickel-sized amount of GloGerm gel was placed in the palm of one hand and applied to both hands completely especially under the nails, around the cuticles and between the fingers before starting the handwashing procedure. The hands were washed using the modified method mentioned above with plain soap and water for 40–60 s and the presence of Glo Germ was observed on the faucet and hands by placing hands under the UV light in a darkened room. A glow under the UV light was considered the presence of contamination.

A self-administered survey questionnaire consisting of one open and seven closed-ended questions (Supplementary Data File) was designed and conducted for the lab staff (*n* = 12) of these two laboratories to assess this handwashing method and determine whether it brought any improvement in their handwashing practices or implementation. The data were analyzed using SPSS v26 and frequencies and percentages were calculated for the survey responses.

## Results

**Figure 2:**
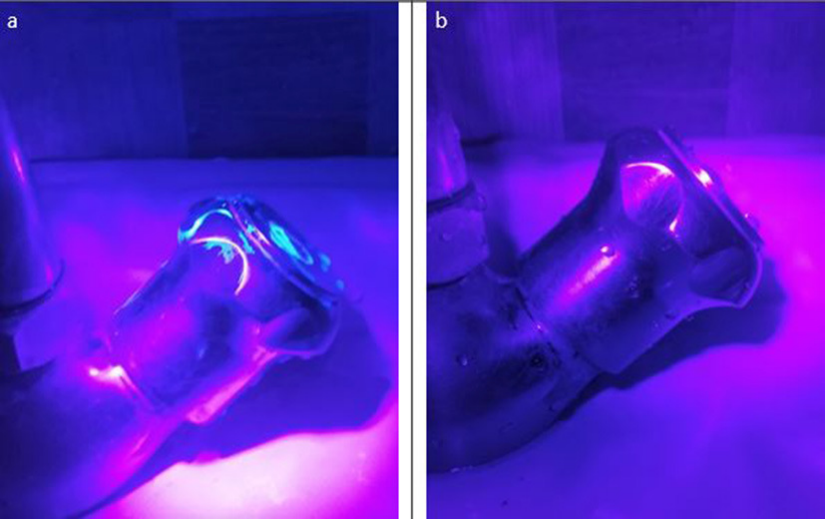
**(a)** Tap is contaminated during step one as visualized by Glo Germ; **(b)** Tap is clean (with no Glo Germ) at the end of the modified hand washing method.

The modified method of handwashing enabled laboratory workers to wash hands with hand/elbow-operated taps without the use of disposable paper towels or tissue papers. With the use of Glo Germ on the hands and faucet, no glow was observed on the faucet or hands at the end of this hand washing method (Figure 2).

Six of the 12 laboratory staff members, surveyed from the two target laboratories, responded. The survey reported that 67% (*n* = 4) of the laboratory professionals working in these laboratories wash their hands 6–10 times daily. The remaining 33% (*n* = 2) wash their hands 1–5 times daily. All (100%) of the survey respondents reported that this method of hand washing is more applicable and implementable in their setting than the WHO handwashing technique that required the use of disposable tissue papers/paper towels. This method enhanced handwashing compliance in their labs, according to 83% (*n* = 5) of laboratory workers. When asked how they used to wash their hands in case of the unavailability of paper towels or tissue papers before the adoption of this method, one person stated that they used to close the faucet with their hands after hand washing and then disinfect hands to remove any germs that remained. Other respondents provided ambiguous responses, stating that they preferred to use hand sanitizers and only washed their hands when required. Eighty three percentage reported that this modified method of handwashing raised handwashing compliance. Eighty three percentage of the lab professionals reported that in their opinion this method adequately disinfects the faucet and the hands of the lab workers.

## Discussion

Laboratory-acquired infections can occur due to varying and suboptimal biosafety practices, and a lack of clear guidance and standard operating procedures (SOPs) (9). Direct contact through contaminated hands is an important mode of transmission of pathogens in laboratories and healthcare settings. Several studies have identified suboptimal hand hygiene as one of the several issues that might have facilitated the occurrence and spread of outbreaks in healthcare facilities (10, 11). Therefore, hand hygiene is considered one of the most significant risk control measures in biosafety. A lot of work has been done to promote hand hygiene across the globe but the global prevalence of washing hands with soap remains at 19%, and the African region's prevalence is even lower at 14% (12). Several studies in Asia including Pakistan reported a baseline hand hygiene compliance between 15 and 66% (13–17). Besides limited awareness of the importance of handwashing and a lack of a culture of biosafety, one important reason could be the lack of sufficient guidance to wash hands in resource-limited settings. No clear guidance is available from USCDC and WHO on how to wash hands when people don't have tissue papers or paper towels in resource-limited settings. A study conducted in Pakistan in 2012 (18) reported that a reusable cloth towel was being used for drying hands after hand washing in several public sector hospitals indicated the unavailability of disposable paper towels and raised questions about how these healthcare workers closed taps after rinsing hands with water to remove foam at the end of handwashing as proposed in the WHO handwashing method. 92.4% of the hospital staff reportedly used the same reusable cloth towel available in a relevant facility despite their dirty condition (86.5%). In this paper, we tried to cover this gap by documenting and implementing a modified handwashing method in which the use of paper towels and towels can be avoided to close the tap at the end of hand washing in two BSL-2 veterinary sector AMR reference laboratories. This doesn't only reduce the cost associated with handwashing but also reduces tissue paper usage making this method eco-friendly by reserving more trees (19). In this method, hands can be air-dried or wiped down with a clean reusable towel after hand washing. The results of this study showed promising outcomes. In this method, soap that is regularly used in laboratories is applied to the faucet for the same amount of time as it takes to wash hands. The use of soap and water for 40–60 min was reportedly enough to remove the majority of the germs that are handled in low-risk laboratories (20, 21). A liquid/solid soap contains chemical agents that have both hydrophilic and hydrophobic properties. One end of these molecules attaches to water while the other end attaches to dirt which is where the bacteria will be. The flow of water at the end of handwashing helps to remove germs from the skin (22) and the faucet. This handwashing method removed the need to apply sanitizer after washing hands, as practiced by some of the survey respondents, to remove any residual germs that can stick to hands by touching dirty faucets and the need for disposable paper towels that is not possible at all times in all resource-limited countries around the world. Hand sanitizers were preferred by the lab workers in these labs, although they are not a cost-effective alternative to hand washing. The preferred use of sanitizers not only reduced handwashing compliance but also increased the laboratory's fiscal burden. The high-risk laboratories may necessitate the use of disposable paper towels and antimicrobial soap as one of the enhanced requirements (23) after a thorough risk assessment in resource-limited settings. The authors believe that this new handwashing method may be in practice informally but has never been documented before. This paper attempted to formally document this method. However, more research is needed to confirm the effectiveness of this hand washing method by increasing the sample size of the study participants and comparing different settings/labs.

## Conclusions

The modified method of handwashing proposed in this paper can improve handwashing compliance in resource-limited settings, where disposable tissue papers are not available. This handwashing method should be validated for practice in resource-limited settings.

## Data availability statement

The original contributions presented in the study are included in the article/Supplementary material, further inquiries can be directed to the corresponding author/s.

## Ethics statement

The studies involving human participants were reviewed and approved by Ethical Review Committee, University of Haripur, Haripur, KP. The patients/participants provided their written informed consent to participate in this study.

## Author contributions

SS: conceptualization, writing—original draft preparation, formal analysis, and project administration. JM: writing—review and editing and methodology. FS: investigation and writing—review and editing. All authors contributed to the article and approved the submitted version.

## Funding

This Project described herein has been funded by UK aid from the UK government through the Fleming Fund Project.

## Conflict of interest

The authors declare that the research was conducted in the absence of any commercial or financial relationships that could be construed as a potential conflict of interest.

## Publisher's note

All claims expressed in this article are solely those of the authors and do not necessarily represent those of their affiliated organizations, or those of the publisher, the editors and the reviewers. Any product that may be evaluated in this article, or claim that may be made by its manufacturer, is not guaranteed or endorsed by the publisher.

## Footnotes

^1^ https://youtu.be/LWmok9avzr4

## References

[1] CDC-NIH. Biosafety in Microbiological Biomedical Laboratories (BMBL). 6th ed. U.S. Department of Health and Human Services and the National Institutes of Health, U.S. Government Printing Office, Washington, DC (2020). p. 1–8.

[2] World Health Organization. Laboratory Biosafety Manual. 4th ed. Geneva: WHO (2020). p. 28.

[3] Mathur P. Hand hygiene: back to the basics of infection control. Indian J Med Res. (2011) 134:611. 10.4103/0971-5916.90985 22199099 PMC3249958

[4] World Health Organization (WHO). Hand Hygiene: Why, How and When? Geneva: WHO (2009). Available online at: https://www.who.int/gpsc/5may/Hand_Hygiene_Why_How_and_When_Brochure.pdf

[5] Ahmed MA Muhammad B Haruna I Abdullahi MA. Pedal based hand washing machine with water level indicator. Int J Multidisc Res Explorer. (2021) 1:74–6. 10.1017/IJMRE.2021406626

[6] Zaied R. Using foot-operated tap mechanism to save water and prevent infection. In: Proceedings of 115th IRES International Conference, Medina, Saudi Arabia, 15th−16th May (2018).

[7] Sarwar S Vijayan V. Pakistan's experience with risk assessment training and implementation of concepts from the 4th edition of the WHO laboratory biosafety manual. J Biosaf Biosecur. (2021) 3:99–107. 10.1016/j.jobb.2021.09.002

[8] World Health Organization. Laboratory Biosafety Manual. 4th ed. and Associated Monographs; Biosafety Program Management. Geneva: WHO (2020).

[9] Byrd JJ Emmert E Maxwell R Townsend H. ASM Task Committee on the Revision of the 2012 laboratory biosafety guidelines. Guidelines for biosafety in teaching laboratories version 2.0: a revised and updated manual for 2019. J Microbiol Biol Educ. (2019) 20:40. 10.1128/jmbe.v20i3.1975 31890075 PMC6914345

[10] Mbouthieu Teumta GM Niba LL Ncheuveu NT Ghumbemsitia MT Itor PO Chongwain P. An institution-based assessment of students' hand washing behavior. Biomed Res Int. (2019) 2019:1–7. 10.1155/2019/7178645 31950052 PMC6949676

[11] Lee MH Lee GA Lee SH Park YH. A systematic review on the causes of the transmission and control measures of outbreaks in long-term care facilities: back to basics of infection control. PLoS ONE. (2020) 15:e0229911. 10.1371/journal.pone.0229911 32155208 PMC7064182

[12] Freeman MC Stocks ME Cumming O Jeandron A Higgins JP Wolf J. Systematic review: hygiene and health: systematic review of handwashing practices worldwide and update of health effects. Trop Med Int Health. (2014) 19:906–916. 10.1111/tmi.12339 24889816

[13] Chhapola V Brar R. Impact of an educational intervention on hand hygiene compliance and infection rate in a developing country neonatal intensive care unit. Int J Nurs Pract. (2015) 21:486–92. 10.1111/ijn.12283 24666764

[14] Chakravarthy M Myatra SN Rosenthal VD Udwadia FE Gokul BN Divatia JV. The impact of the International Nosocomial Infection Control Consortium (INICC) multicenter, multidimensional hand hygiene approach in two cities of India. J Infect Public Health. (2015) 8:177–86. 10.1016/j.jiph.2014.08.004 25270387

[15] Khan A Nausheen S. Compliance of surgical hand washing before surgery: role of remote video surveillance. J Pak Med Assoc. (2017) 67:92–6. 28065962

[16] Mu X Xu Y Yang T Zhang J Wang C Liu W. Improving hand hygiene compliance among healthcare workers: an intervention study in a hospital in Guizhou Province, China. Braz J Infect Dis. (2016) 20:413–18. 10.1016/j.bjid.2016.04.009 27351752 PMC9425469

[17] Su D Hu B Rosenthal VD Li R Hao C Pan W. Impact of the International Nosocomial Infection Control Consortium (INICC) multidimensional hand hygiene approach in five intensive care units in three cities of China. Public Health. (2015) 129:979–88. 10.1016/j.puhe.2015.02.023 25818015

[18] Rao MH Arain GM Khan MI Talreja KL Ali G Munir MK. Assessment of knowledge, attitude and practices pattern of hand washing in some major public sector hospitals of Pakistan (a multi-center study). Pak J Med Res. (2012) 51:76A.

[19] Simple Ecology,. Soft Tissue Paper Is Hard on the Environment. (2014). Available online at: https://www.simpleecology.com/blog/soft-tissue-paper (accessed May 16, 2022).

[20] Safety WP. WHO Guidelines on Hand Hygiene in Health Care. Geneva: WHO (2009).

[21] Wise J. Ordinary soap is as effective as antibacterial soap for handwashing, study finds. BMJ. (2015) 351:h4944. 10.1136/bmj.h4944 26377850

[22] Rogers E,. How Does Handwashing Kill Germs? (2020). Available online at: https://cnylatinonewspaper.com/english/how-does-handwashing-kill-germs/ (accessed April 1, 2022).

[23] Stewardson AJ Pittet D. Hand Hygiene. A Guide to Infection Control in the Hospital. 5th ed. Brookline, MA: International Society for Infectious Diseases (2014). p. 22–30.

