# Supplementary material for: A modified hand washing method for resource limited settings
Source: Front Public Health. 2022 Aug 4;10:965853. doi: 10.3389/fpubh.2022.965853 (PMC9386356; doi:10.3389/fpubh.2022.965853)
Supplement: Supplementary file 1 [file Table_1.pdf]

Supplementary Table. Handwashing SOP Implementation Follow-up Survey

**Question**	**Options**				
On average how many times do you wash your hands in a day while working in a lab?	1 - 5	6 – 10	11 - 15	16 - 20	More than 20
Did you read the hand washing SOP that was developed for your lab?	Yes	No			
What would be the best way to communicate this modified handwashing technique to lab workers?	SOP as it is	Pictures stuck visibly at the handwashing sink	Repeated verbal communication		
Is this method of hand washing more applicable and implementable than the WHO hand washing technique that required the use of tissue papers?	Yes	No			
Did this SOP improve compliance with hand washing in your lab?	Yes	No			
Before the development of this SOP, how you used to wash your hands in case of the unavailability of paper towels or tissue paper?	Open-ended question				
Was the WHO method that required the use of tissue papers a hindrance in hand washing practices in your lab?	Yes	No	Somewhat	A great deal	
Do you think that this method adequately disinfects the taps and the hands of the lab workers?	Yes	No	May be		
